# From cat scratch disease to endocarditis, the possible natural history of *Bartonella henselae *infection

**DOI:** 10.1186/1471-2334-7-30

**Published:** 2007-04-18

**Authors:** Frédérique Gouriet, Hubert Lepidi, Gilbert Habib, Frédéric Collart, Didier Raoult

**Affiliations:** 1 Unité des Rickettsies, CNRS UMR 6020, IFR 48, Faculté de Médecine, Université de la Méditerranée, 27 Boulevard Jean Moulin, 13385 Marseille cedex 05, France; 2 Service de Cardiologie B, Centre Hospitalier Universitaire de La Timone, Marseille, 247, rue Saint-Pierre, Marseille 13385 Cedex 5, France; 3 Service de Chirurgie Cardiaque, Centre Hospitalier Universitaire de La Timone, 247, rue Saint-Pierre, Marseille 13385 Cedex 5, France

## Abstract

**Background:**

Most patients with infectious endocarditis (IE) due to *Bartonella henselae *have a history of exposure to cats and pre-existing heart valve lesions. To date, none of the reported patients have had a history of typical cat scratch disease (CSD) which is also a manifestation of infection with *B. henselae*.

**Case presentation:**

Here we report the case of a patient who had CSD and six months later developed IE of the mitral valve caused by *B. henselae*.

**Conclusion:**

Based on this unique case, we speculate that CSD represents the primary-infection of *B. henselae *and that IE follows in patients with heart valve lesions.

## Background

*Bartonella *spp. are link to the host immune system, infection with the same *Bartonella *species (*e.g., B. henselae*) can result in focal suppurative reaction (cat scratch disease in immunocompetent patients), a multifocal angioproliferative response (bacillary angiomatosis in immunocompromised patients), an increased immune response without evidence of bacteria in patient tissues (meningoencephalitis), or endovascular multiplication of the bacteria (endocarditis). Of the 19 species within the genus *Bartonella *[1], seven are known to cause infective endocarditis (IE) in people: *B. quintana *[2], *B. henselae *[3], *B. elizabethae *[4], *B. vinsonii *subps. *berkhoffii *[5], *B. vinsonii *subps. *arupensis *[6], *B. kohlerae *[7] and *B. alsatica *[8]. These zoonotic agents cause 1 to 15% of all cases of IE [3,9,10] and cannot be detected using routine blood cultures due to the fastidious nature of the bacteria. The most widely used method for the laboratory diagnosis of infection with *Bartonella *sp. is serology. Enzyme immunoassay (EIA) has been described [11] but indirect immunofluorescence antibody assay (IFA) is the reference technique [12], despite cross-reactivity among *Bartonella *spp. [13]. Our laboratory uses two IFA titers in the diagnosis of *Bartonella *infections. In conjunction with a compatible medical history, an IgG titer ≥1:50 to *B. henselae *suggests a diagnosis of acute infections such as cat scratch disease (CSD), while IgG ≥1:800 to either *B. henselae *or *B. quintana *suggests a diagnosis of endocarditis [14,15]. Western-blotting with adsorbed sera enables us to differentiate between infections with *B. henselae *and *B. quintana *[13]. Currently, the literature describes *B. henselae *as an agent causing a typical endocarditis which is easily diagnosed using the Duke criteria [3,16] and usually with vegetations that can be detected by echocardiography. Patients usually have a pre-existing cardiac valve lesion and although they are exposed to cats, they usually do not have a history of CSD. Here we report a patient who suffered from CSD and subsequently developed *B. henselae *IE.

## Case presentation

### Case

**Figure 1:**
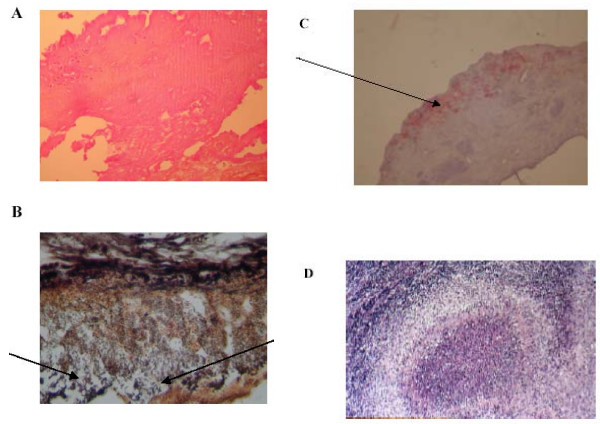
**Valve of our patient with *B. henselae *endocarditis**. Resected valve with *B. henselae *infection showing large and non-inflammatory vegetation on the valvular surface (A, hematoxylin-eosin, original magnification × 100). The diagnosis of vegetation was made by the presence of fibrinous material with numerous darkly stained bacilli (arrows in Figure 1B) consistent with *Bartonella*, organized in numerous clusters (B, Warthin-Starry silver staining, original magnification × 400). The bacteria (arrows in Figure 1C) are detected by immunohistochemical analysis in an extracellular location inside the valvular vegetation (C, polyclonal antibody anti-*B. henselae *with hematoxylin counterstain, original magnification × 200). Resected lymph node showed a necrotizing lymphadenitis. Numerous microabscesses composed of fragmented neutrophils were observed in homogenous necrotic areas. Necrotic regions were surrounded by a ring of macrophages and epithelioid histiocytes to form stellate inflammatory granulomas consistent with cat-scratch disease (D, hematoxylin-eosin, original magnification × 100).

**Figure 2:**
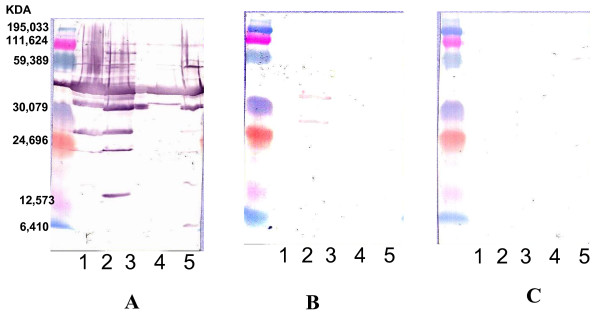
**Western blotting using *Bartonella *antigen of a patient with IE**. Western blotting was performed with the first serum sample from May 2005 at a 1:200 dilution. Molecular masses (in kilodaltons) are given on the left. A through C: Serum was analyzed by using *B. quintana *(lane 1), *B. henselae *(lane 2), *B. elizabethae *(lane 3), *B. vinsonii *subsp. *Berkhoffii *(lane 4) and *B. alsatica *(lane 5) antigens. (A) Untreated serum. (B) *B. quintana*-adsorbed serum. Antibodies to the *B. henselae *remained. (C) *B. henselae-adsorbed *serum. All antibodies were removed.

In May 2005, a 43-year-old man was admitted to the hospital with mitral regurgitation. In 1981, he had been in a car crash and developed a destructive nosocomial *Staphylococcus aureus *endocarditis of the mitral valve. A bioprosthesis was inserted which failed in 1988 and was replaced. In May 2005, regurgitation through the valve was once again detected and the patient was hospitalized for a further valve replacement. The patient was afebrile and had a systolic murmur over the mitral area. He had no leukocytosis (leukocyte count was 3.63 × 10^9^/l with 50.2% neutrophils). The low neutrophil count corrected itself spontaneously. The erythrocyte sedimentation rate (16/43 mm) and C-reactive protein (<5 mg/l) was normal and hepatic enzymes were elevated (ALT: 69 IU/L; normal ≤ 40 IU/L). Three routine blood cultures were negative (Bactec, Becton Dickinson, Sparus, Maryland) and no rheumatoid factor was detected. Transthoracic echocardiography revealed mitral insufficiency but there were no vegetations and IE was not considered as a possible diagnosis. However, no transesophageal echocardiography was performed. Histology of the prosthetic valve removed at surgery using reported methods [17,18], revealed an IE with a vegetation containing micro-organisms that stained with Warthin-Starry and Giemsa [19] (Figure 1). Standard cultures of cardiac valve tissue remained sterile, but with cell-cultures (human endothelial cell) a strain of *B. henselae *was isolated in 3 weeks [20]. Also, DNA of *B. henselae *was demonstrated to be present in the valve by PCR and sequencing with primers for the eubacterial 16S rRNA gene [21] and *Bartonella *ITS region [3] Genotyping of the *B. henselae *strain was carried out using the multi-spacer typing (MST) method as previously described [22]. Sequences obtained from the nine studied spacers classified the strain within MST genotype five, previously described to contain cat isolates from various countries including France, Germany and USA [22]. Serum tested retrospectively was found to contain antibodies to *B. henselae *and *B. quintana *at an IgG titer of 1:200 [14], which is not suggestive of IE. However, western blotting was positive for antibodies to *B. henselae *and *B. quintana *and showed a reactivity pattern typical for endocarditis [13] (Figure 2). Immunoblotting with a serum sample adsorbed with *B. henselae *confirmed the diagnosis of *B. henselae *IE.

The diagnosis of IE was made retrospectively based on the combination of histology of the cardiac valve lesions, culture of *Bartonella *from the valve, presence of a predisposing heart condition, and serological evidence of *Bartonella *infection. Without the histology of the valve the patient would not have had a positive score using the Duke criteria; he would only have had 2 minor criteria. After surgery, the patient recovered rapidly with routine post-surgical amoxicillin administration for 4 days, followed by gentamycin for 15 days and doxycycline for 1 month [23].

Retrospectively, it was found that six months before the patient had had suspected lymphoma of an inguinal lymph node. Histology of the node, however, showed a necrotizing lymphadenitis suggested of CSD. Numerous microabscesses containing fragmented neutrophils were observed in homogenous necrotic areas. These necrotic regions were surrounded by a ring of macrophages and epithelioid histiocytes to form stellate inflammatory granulomas (Figure 1). No bacteria were detected by immunohistochemical examination or Warthin-Starry staining. Unfortunately, the lymph node sample was not available for PCR analysis to confirm the diagnosis of CSD.

The patient did not own a cat but reported a single contact with a stray cat that scratched him one month before the enlargement of the inguinal lymph node. We report the development of IE after a likely episode of CSD in a patient with a mechanical mitral cardiac valve. In previous studies [16], *B. henselae *was described in patients who have regular contact with cats and with pre-existing valvulopathies [24,25], but to the best of our knowledge the progression of CSD to IE has not previously been reported.

## Discussion

We describe here the development of IE six months after CSD in a patient with a known valvulopathy. The diagnosis of CSD was histological as blood collected at the time was not available for serological or DNA testing. Serology in May 2005, however, revealed a titer of 1:200 against the *Bartonella *antigens, which is intermediate between the titer we use to diagnose CSD and that for IE [26]. Most patients with *Bartonella *IE have high titers >800 [14]. However, in our case the western-blotting showed an endocarditis profile (Figure 2) and cross adsorption confirmed the diagnosis of *B. henselae *infection [13]. Also, *B. henselae *genotype five was identified by both PCR and culture of the cardiac valve. The possibility of chronic bacteremia due to *B. henselae *has been reported in a patient during the natural course of CSD [27]. The isolation of the *Bartonella *species is difficult, especially in immunocompetent patients, and cultures of clinical samples are most often sterile. The best specimens for PCR amplification are cardiac valve biopsies, but these are seldom available. Testing can be performed even following antibiotic therapy. Six months after our patient likely suffered from CSD, we diagnosed asymptomatic IE with degenerative lesions in the valve but no vegetations were found echocardiographically.

Therefore, the natural history of *B. henselae *may resemble that of Q fever [28]. As with *B. henselae*, the majority of patients with Q fever IE have pre-existing valve lesions [16]. Between 30 and 50% of patients with cardiac valve lesions that have primary infections with *Coxiella burnetii *(symptomatic or asymptomatic) develop IE within 2 years [29]. However, the delay between bacteremia and IE ranges from months to years and we now recommend that the presence of valve lesions be carefully investigated in patients with Q fever as this enables early treatment [30].

**Figure 3:**
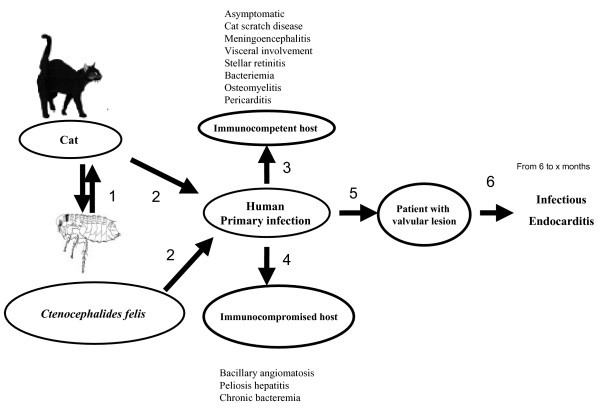
**Natural history of *B. henselae *endocarditis**. 1: Cycle of *B. henselae *between cat and *Ctenocephalides felis*. 2: Contamination of human being. 3: Clinical presentation in non immunocompromised patient with contact with cat. 4: Clinical presentation contact in immunocompromised with contact with cat. 5: Predisposing vavular lesion. 6: Endocarditis.

In various studies, the seroprevalences of antibodies to *B. henselae *in healthy people have ranged from 3 to 6% [31]. This suggests that many primary infections with *B. henselae *are asymptomatic [32]. Asymptomatic primary infections may result in IE as well. We speculate that following exposure to *B. henselae*, a patient may develop bacteremia with or without clinical signs of typical CSD [27]. In patients with valvular lesions this may result in IE. In our patient, a diagnosis of IE was not considered as the condition was asymptomatic and it may have been several months before typical clinical signs would have become apparent (Figure 3). If our hypothesis is confirmed, it should become routine for patients with CSD to be examined for valve lesions with echocardiography. While antibiotic treatment is not currently recommended for patients with CSD [23], treatment might be indicated in patients who have valve lesions as this might prevent IE as has been reported for patients with Q fever [29]. IE is a life threatening disease and it is critical that a diagnosis be made as early as possible. We suggest that patients with CSD and valvular heart disease should be tested within a year for serological, blood culture or DNA detection of continued *B. henselae *infection [1]. If these tests are positive, early antibiotic treatment may be indicated to prevent IE.

## Conclusion

This single case report provides further insight into the natural history of *B. henselae *IE. To our knowledge this is the first report of the progression of probable CSD to *B. henselae *IE in a patient with a pre-existing cardiac valve lesion and only a single contact with a cat. It shows that contact with a cat and CSD are risk factors for IE in patients with cardiac valve disease. Our findings suggest echocardiography may be indicated in patients with CSD. Follow-up of patients with cardiac valve lesions that develop CSD may enable the early treatment of *B. henselae *IE.

## Competing interests

The author(s) declare that they have no competing interests.

## Authors' contributions

FG participated in the conception of the study, participated in its coordination, drafted the paper and took care of the hospitalized patient. HL performed anatomopathology and immunohistochemistry. GH took charge of the patient and followed the patient. FC carried out the surgery. DR conceived the study, coordinated it, finalized the paper and followed the patient after hospitalization. All authors read and approved the final manuscript.

## Pre-publication history

The pre-publication history for this paper can be accessed here:


